# Comprehensive analysis of circRNA-miRNA-mRNA network related to angiogenesis in recurrent implantation failure

**DOI:** 10.1186/s12920-024-01944-1

**Published:** 2024-07-30

**Authors:** Anran Wang, Piaopiao Chen

**Affiliations:** 1 Department of Adult Chinese Medicine, Qingdao Women and Children’s Hospital, Tongfu Road, Qingdao, Shandong 266034 China; 2 Department of Orthopedics 1, Qingdao Huangdao District Second Traditional Chinese Medicine Hospital, Zhongyuan Street, Qingdao, Shandong 266427 China

**Keywords:** Recurrent implantation failure, Endometrial receptivity, Circular RNA, Regulatory network, Angiogenesis, Signaling pathway, Molecular pathogenesis

## Abstract

**Background:**

Abnormal endometrial blood flow causes a decrease in endometrial receptivity and is considered a relatively independent risk factor for recurrent implantation failure (RIF). This study aimed to explore the potentially functional circRNA-miRNA-mRNA network in RIF, and further explore its mechanism.

**Methods:**

Datasets were downloaded from the GEO database to identify differentially expressed circRNAs, miRNAs and mRNAs. The circRNA–miRNA–mRNA and PPI networks were constructed using Cytoscape 3.6.0 and the STRING database, the hub genes were identified with the cytoHubba plug-in, and a circRNA–miRNA–hub mRNA regulatory sub-network was constructed. Then, GO and KEGG pathway enrichment analyses of the hub genes were performed to comprehensively analyze the mechanism of hub mRNAs in RIF. Due to the results of circRNAs-miRNAs-hub mRNAs regulatory network, we verified the expression of circRNA_0001721, circRNA_0000714, miR-17-5p, miR-29b-3p, HIF1A and VEGFA in the RIF mouse model by qRT‒PCR and western blotting.

**Results:**

We initially identified 175 DEmRNAs, 48 DEmiRNAs and 56 DEcircRNAs in RIF associated with angiogenesis and constructed a circRNA-miRNA‒mRNA network and PPI network. We further identified six hub genes in the acquired network. Based on these genes, functional enrichment analysis revealed that the HIF-1 signaling pathway plays a vital role in endometrial angiogenesis in RIF. In addition, the interaction networks of circRNA_0001721/miR-17-5p/HIF1A and the circRNA_0000714/miR-29b-3p/VEGFA axis were predicted. In the RIF mouse model, circRNA_0001721, circRNA_0000714, HIF1A and VEGFA were down-regulated, whereas miR-17-5p and miR-29b-3p were up-regulated according to qRT‒PCR and western blotting.

**Conclusion:**

This study revealed that the HIF-1 signaling pathway plays a vital role in endometrial angiogenesis in RIF. The circRNA_0001721/miR-17-5p/HIF1A and circRNA_0000714/miR-29b-3p/VEGFA axes might play a role in the pathogenesis of endometrial angiogenesis in RIF.

**Supplementary Information:**

The online version contains supplementary material available at 10.1186/s12920-024-01944-1.

## Introduction

Recurrent implantation failure (RIF) refers to the failure to achieve embryo implantation after two or more consecutive embryo transfer cycles or freeze‒thaw embryo transfer (FET) cycles, where the cumulative number of transferred embryos is not less than four high-quality cleavage-stage embryos or not less than two blastocysts [1]. Successful pregnancy depends on high-quality embryo quality and good endometrial receptivity. In the FET cycle, even with the highest quality blastocysts, the endometrium must be selective and acceptable [2]. However, two-thirds of implantation failures are believed to be secondary to poor endometrial receptivity [3].

Decreased endometrial receptivity is considered one of the most important factors in RIF [4]. The blood perfusion of the microenvironment at the embryo implantation site is related to the receptivity of the endometrium. Adequate blood perfusion usually indicates that the blood vessels are rich in distribution and that the receptivity of the endometrium is good [5]. Sardana et al. reported an association between pregnancy rates and subendometrial blood flow in hormonal replacement FET cycles [6]. A number of studies have shown that the vascularization flow index (VFI) on the day of transplantation is an independent factor affecting posttransplantation pregnancy [7], which indicates that promoting endometrial angiogenesis can improve endometrial receptivity [8] and thus improve the final outcome of patients with RIF.

Circular RNAs (circRNAs) are a special new class of endogenous noncoding RNAs that are covalently closed single-stranded circular RNA molecules formed by backsplicing [9]. CircRNAs are abundant in eukaryotic cells and have been confirmed to be specific to tissue, developmental stage and disease [10]. Researchers have shown that circRNAs can regulate gene expression in mammals by sequestering microRNAs (miRNAs) as miRNA sponges with miRNA response elements, directly binding with RNA-binding proteins, and even translating proteins [11]. Researchers have shown that circRNAs play important roles in cancer through the circRNA-miRNA‒mRNA regulatory axis, thus affecting the occurrence and development of cancer [12]. Many studies have shown that circRNAs can regulate the occurrence and development of different kinds of diseases [13–16]. Liu et al. reported altered circular RNA expression in patients with RIF [17] However, whether some circRNAs can act as miRNA sponges to affect endometrial angiogenesis has not been reported.

**Fig. 1:**
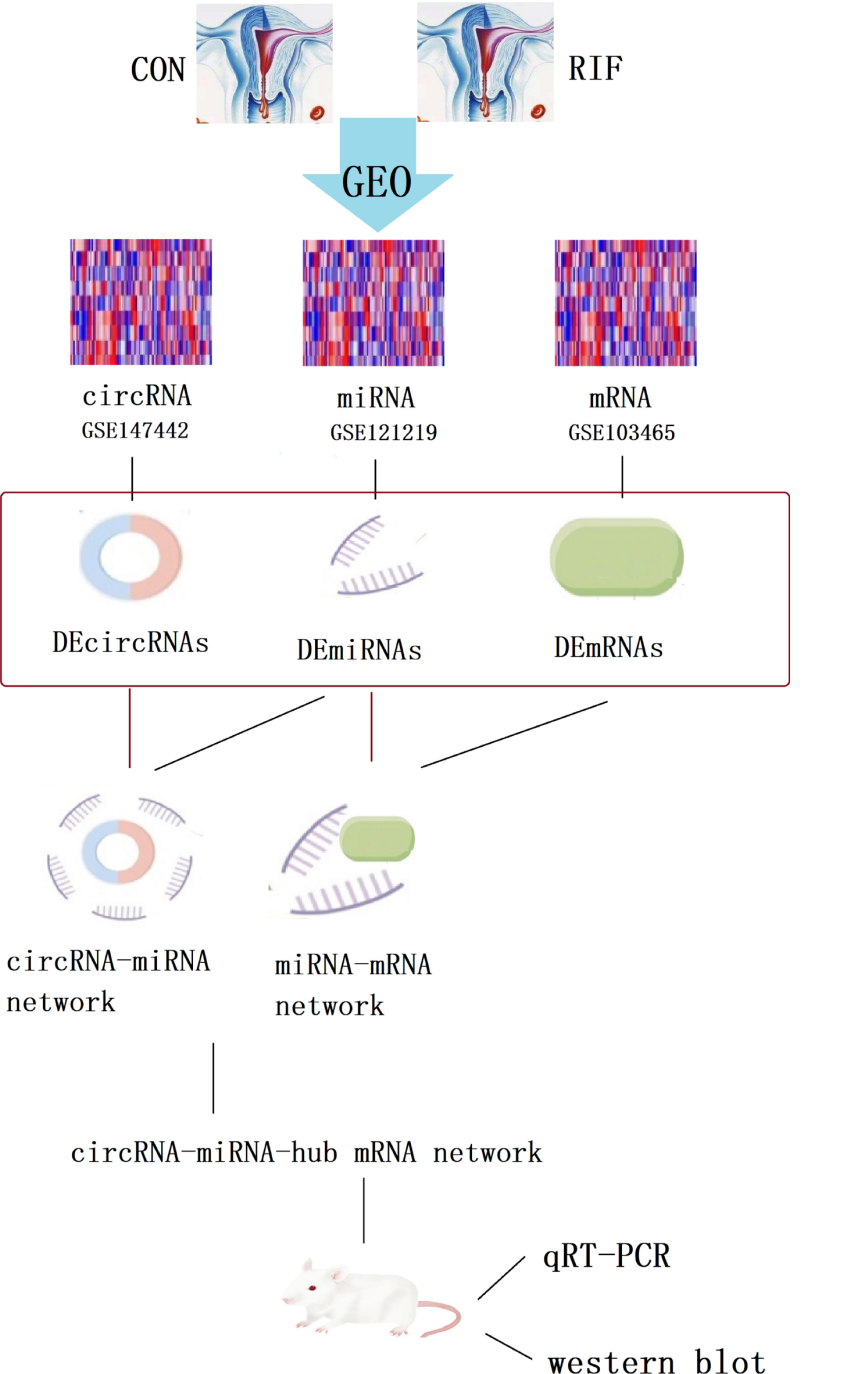
Study design flowchart. circRNA, circular RNA; miRNA, microRNA; RIF, recurrent implantation failure. DEcircRNAs, differentially expressed circRNAs; DEmiRNAs, differentially expressed miRNAs; DEmRNAs, differentially expressed mRNAs

In this study, we established circRNA-miRNA-mRNA regulatory network which was involved in the process of endometrial angiogenesis in RIF and provided insight into the underlying mechanism and treatment strategy of RIF. The workflow is shown in Fig. 1.

## Materials and methods

### Data extraction

CircRNA, miRNA and mRNA microarray expression profile datasets were selected from the National Center of Biotechnology Information Gene Expression Omnibus (NCBI) GEO (https://www.ncbi.nlm.nih.gov/geo/).

The circRNA microarray data were retrieved from GSE147442, which is based on the GPL21825, 074 301 Arraystar Human CircRNA Microarray v2. GSE147442 included 8 pairs of RIF patients and controls’ endometrial tissue which were used to screen for DEcircRNAs.

The miRNA microarray data were derived from the GEO database in GSE121219, which was based on the GPL18058 Exiqon miRCURY LNA microRNA array, 7th generation [miRBase v18, condensed Probe_ID version]. This database was from 8 RIF patients and 10 matched controls’ endometrial tissue.

The mRNA microarray data were extracted from the GSE103465 dataset, which is based on the GPL16043 GeneChip^®^ PrimeView™ Human Gene Expression Array (with external spike-in RNAs). There were screened from 3 pairs of patients with RIF and controls’ endometrial tissue in the GSE103465 dataset.

All samples tested were endometrial tissue at the implantation window. The raw microarray datasets were preprocessed by background correction and normalization in the GEO database.

### Screening out DERNAs

The DEcircRNAs, DEmiRNAs and DEmRNAs between RIF patients and controls were identified by analysis with GEO2R in the GEO database. Adjusted P values < 0.05 and |log2FC|>1 were used as thresholds for the screening criteria of DEcircRNAs, DEmiRNAs and DEmRNAs. Finally, differentially expressed mRNAs (DERNAs), including DEcircRNAs, DEmiRNAs and DEmRNAs, were visualized by volcano plots.

### Prediction of angiogenesis-related DEmRNAs

Angiogenesis-related mRNAs were obtained from GeneCards (https://www.genecards.org/), which is an integrative database that provides all known human genes from sources such as genomic, transcriptomic, proteomic, genetic, clinical and functional information. Angiogenesis-related mRNAs obtained from GeneCards were filtered by a relevance score ≥ 1. Then, the angiogenesis-related mRNAs retrieved from the GeneCards database and the DEmRNAs from the GSE103465 dataset were mapped in jvenn [18] (http://bioinfo.genotoul.fr/jvenn.) to obtain the overlapping angiogenesis-related DEmRNAs.

### Prediction of angiogenesis-related miRNA‒mRNA pairs

DEmiRNA target genes were predicted by the microRNA Data Integration Portal (miRDIP, http://ophid.utoronto.ca/mirDIP/index.jsp), which integrates more than 20 miRNA-related databases for miRNA target prediction [9]. Next, among the DEmRNAs related to angiogenesis, potential DEmiRNA targets that had a very high score (top 1%) in the miRDIP were selected. Then, the potential DEmiRNA targets in the miRDIP and the DEmiRNAs from the GEO database were mapped in jvenn to obtain the overlapping angiogenesis-related DEmiRNAs.

### Prediction of angiogenesis-related circRNA-miRNA pairs

The DEmiRNA-targeted circRNAs were predicted by starBase 2.0 (https://starbase.sysu.edu.cn/index.php), which is a database developed for deciphering protein‒RNA and miRNA‒target interactions, such as miRNA‒lncRNA, miRNA‒mRNA, miRNA-circRNA, miRNA-pseudogene, miRNA-sncRNA interactions and ceRNA networks from 108 CLIP-Seq (HITS-CLIP, PAR-CLIP, iCLIP, CLASH) datasets [19] and refined results with high stringency of CLIP Data: 3. Then, the predicted circRNAs and the DEcircRNAs from the GEO database were mapped in jvenn to obtain the overlapping angiogenesis-related DEcircRNAs.

### Construction of the circRNA-miRNA‒mRNA network related to Angiogenesis

After obtaining the intersection of circRNA-miRNA and miRNA‒mRNA pairs, a circRNA-miRNA‒mRNA network related to angiogenesis was constructed and visualized using Cytoscape 3.6.0.

#### Identification of hub genes in the CircRNA-miRNA‒mRNA Regulatory Network

Based on the DEmRNAs, a protein‒protein interaction (PPI) network was constructed with STRING (https://cn.string-db.org/). Next, the PPI network data were input into and visualized with Cytoscape 3.6.0. Finally, the degree, betweenness centrality and closeness centrality of the DEmRNAs were determined via network analysis in Cytoscape 3.6.0 and used to identify the RIF angiogenesis-related hub genes via the cytoHubba plug-in.

### Gene ontology and kyoto encyclopedia of hub DEmRNAs

Gene Ontology (GO) analysis and Kyoto Encyclopedia of Genes and Genomes (KEGG) pathway enrichment analyses of the hub genes were performed by Metascape (https://Metascape.org/gp/index.html), which combines feature-rich and interactive group analysis, utilizing over 40 independent knowledge bases for comprehensive analysis within an integrated portal [20]. Both the *P* values of the GO analysis and KEGG analysis were < 0.05.

### Experimental mouse model of RIF

The study was reported in accordance with Animals in Research: Reporting In Vivo Experiments (ARRIVE) guidelines. The study protocol was reviewed and approved by the ethics committee of the Medical College of Qingdao University (QDU-AEC-2023127). Using resource equation approach [21], we calculated that the sample size of this test was 7 to 13, considering the funding for the experiment, we choose ten 6- to 8-week-old ICR female mice were randomly divided into control (*n* = 5) and model (*n* = 5) groups. After one week of adaptive feeding, the bedding of the male and female mice changed, and 1–2 days later, the female mice underwent visual observation combined with vaginal smear analysis to determine their oestrus status. If the smear is stained with Giemsa and observed under a microscope, all nonnucleated keratinized epithelial cells are classified as oestrus. Afterwards, the estrous ICR female mice and male mice will be mated at a 2:1 ratio, which will be recorded as the 0th day of pregnancy.

Due to the high sensitivity of the endometrium to mifepristone [22], administering mifepristone at this time can prevent the transition of the endometrium from the proliferative phase to the secretory phase before implantation, leading to a decrease in endometrial receptivity and inhibition of implantation [23]. Subcutaneous injection of the lowest effective dose of mifepristone in pregnant mice can establish a stable animal model of embryo implantation disorders [24]. Mifepristone tablets were prepared in an oil solution at a concentration of 0.68 mg/mL using sesame oil. On the 4th day of pregnancy, the control group was subcutaneously injected with 0.1 mL of sesame oil, while the model group was subcutaneously injected with 0.1 mL of mifepristone oil.

On the afternoon of the 5th day of pregnancy, the mice in each group were anesthetized with 3% solution of pentobarbital sodium with sterile physiological saline at the usual dose of 30 mg/kg body weight by intraperitoneal injection. During the injection process, the space was intermittently and slowly. After injection to 3/4 of the predetermined dose, proceed more slowly while observing the animal’s corneal reflex, muscle relaxation, and pain response until reaching the required anesthesia state for the experiment. When the anesthesia required for the experiment was reached, immediately stopped the injection of the drug. Then the mice were sacrificed by cervical dislocation. The uterine morphology of the mice was observed with the naked eye. The number of embryos implanted was significantly lower in the model group than in the control group, indicating that mifepristone oil was used to induce the RIF mouse model.

### Quantitative real-time polymerase chain reaction (qRT‒PCR)

We extracted the endometrium from each female mouse at the implantation site of the blastocyst and homogenized the endometrial tissue using lysis buffer and a homogenizer to completely separate the nucleic acid protein complex. According to the manufacturer’s protocol, total RNA was extracted from endometrial tissue using TRIzol reagent (Ambion, USA). An M5 Sprint qPCR RT kit (Mei5 Biotech, Beijing, China) with gDNA remover and a miRNA first strand cDNA synthesis kit (Servicebio, Wuhan, China) were used to reverse transcribe 1 mg of RNA from each sample into cDNA. According to the standard protocol, qPCR was performed using 1x Hieff qPCR SYBR Green Master Mix (Servicebio, Wuhan, China) on a CFX96 Real-Time System (Bio-Rad, USA). Specific primers for mRNA, miRNA, and circRNA were synthesized by Sangon Biotech (Shanghai, China). qRT‒PCR SYBR green assay sequences were shown in Table 1.

The relative transcription levels of circRNAs and mRNAs were determined by β-actin standardization, and U6 was used as an internal control for miRNAs. The expression levels of each mRNA, miRNA, and circRNA were calculated using the 2^−ΔΔCt^ method.

**Table 1:** qRT‒PCR SYBR green assay sequences

Name	Sequence (5′→3′)
mmu-circ-0000714 F	ACTACTCGTCTGAGCAGGGT
mmu-circ-0000714 R	AGGGTTTTTCTTGCATAACTGC
mmu-circ-0001721 F	CTGTCCTCATTTGCCCCTGT
mmu-circ-0001721 R	GGAGAGCTTTGCTACCCTCC
M-GAPDH F	CCTTCCGTGTTCCTACCCC
M-GAPDH R	GCCCAAGATGCCCTTCAGT
miR-29b-3p F	TAGCACCATTTGAAATCAGTGTT
miR-17-5p F	CAAAGTGCTTACAGTGCAGGTAG
M-U6 F	CTCGCTTCGGCAGCACATATACT
M-U6 R	ACGCTTCACGAATTTGCGTGTC
VEGFA F	CTACTGCCGTCCGATTGAG
VEGFA R	TGCTGGCTTTGGTGAGGTTT
HIF1A F	CTCCTGTAAGCAAGGAGCCA
HIF1A R	ACATTGTGGGGAAGTGGCAA
M-GAPDH F	CCTTCCGTGTTCCTACCCC
M-GAPDH R	GCCCAAGATGCCCTTCAGT

### Western blot

We placed mouse endometrial tissue on ice and lysed it in RIPA lysis buffer to extract total protein. The processed samples were quantified according to 20 µg of total protein on 8-12% SDS polyacrylamide gels for electrophoresis for 1 h and then transferred to 0.2 at 4 °C. The proteins were incubated on a PVDF membrane (Bio-Rad) in 5% skim milk powder for 1 h. Primary antibodies were prepared with 5% volume fraction skim milk powder (VEGFA, mouse monoclonal antibody, Servicebio Company) at a dilution of 1:1000, HIF-1 A (mouse monoclonal antibody, Servicebio Company) at a dilution of 1:2000, and β-actin (internal reference, mouse monoclonal antibody, Proteintech Company) at a dilution of 1:2500. After the cells were washed 5 times with 1× PBS (5 min each), they were incubated with horseradish peroxidase-labeled goat anti-mouse IgG (1:5000) for 40 min before being washed. The protein expression level was observed by chemiluminescence, and the relative grayscale value was calculated and compared with that of the control group using ImageJ software.

### Statistical analysis

Data were expressed as means ± SD. Statistical analysis was performed using SPSS (25.0).Student’s paired t-test analyses were performed in two groups. The signifcant diferences were considered when *P* < 0.05.

## Results

### Identifying DEmRNAs associated with angiogenesis

**Fig. 2:**
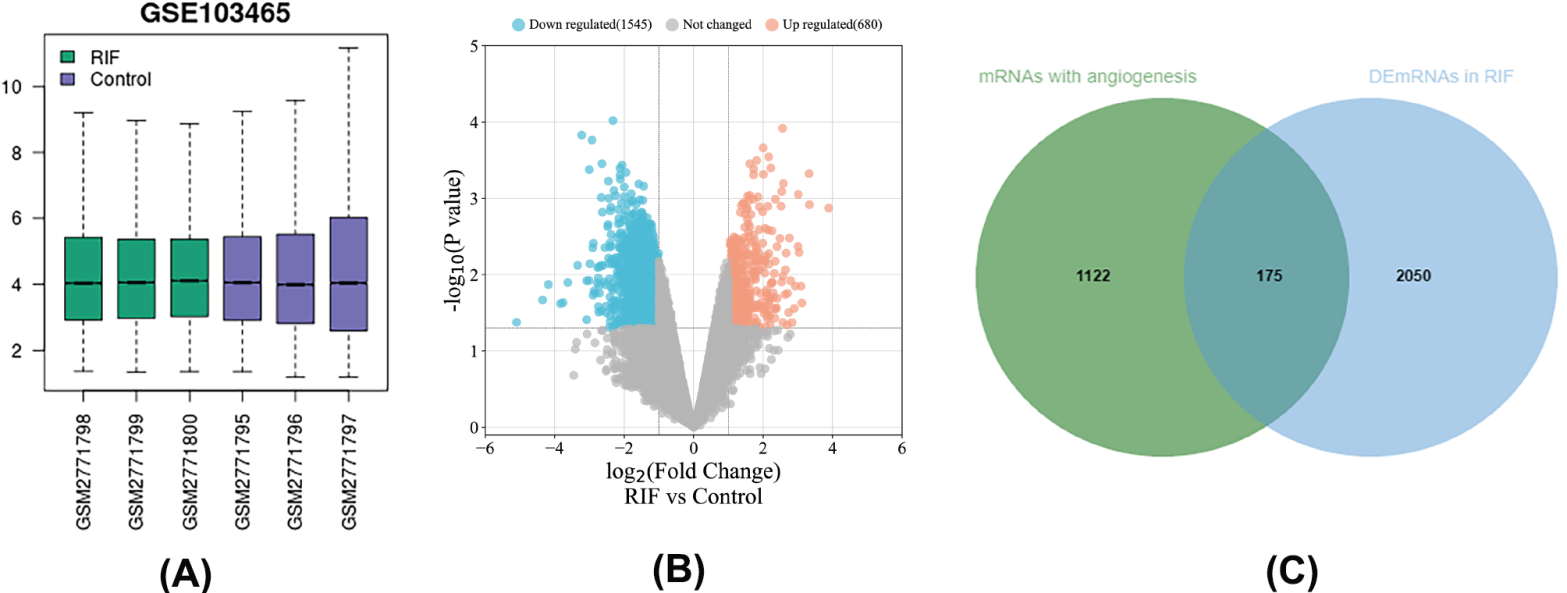
Boxplots, volcano plots and Venn diagram of mRNAs for GSE103465. (A) Boxplot of GSE103465. (B) Volcano plots of DEmRNAs based on GSE103465. (C) Venn diagram of mRNAs for GeneCards and GSE103465, where the intersection section is predicted DEmRNAs asscociated with angiogenesis in RIF. circRNA, circular RNA; miRNA, microRNA; RIF, recurrent implantation failure

Two thousand, two hundred and twenty-three DEmRNAs (680 up-regulated and 1545 down-regulated) were screened from endometrial tissues from 3 paired RIF patients and controls at the implantation window in GSE103465 (Fig. 2A and B, Supplementary Table 1). One thousand, two hundred and ninety-seven DEmRNAs were found to be associated with angiogenesis according to the relevance score in GeneCards (Supplementary Table 2). Finally, one hundred and seventy-five DEmRNAs associated with angiogenesis were obtained after taking the intersection of the results from GeneCards and GEO (Fig. 2C).

### Prediction of angiogenesis-related miRNA‒mRNA pairs

**Fig. 3:**
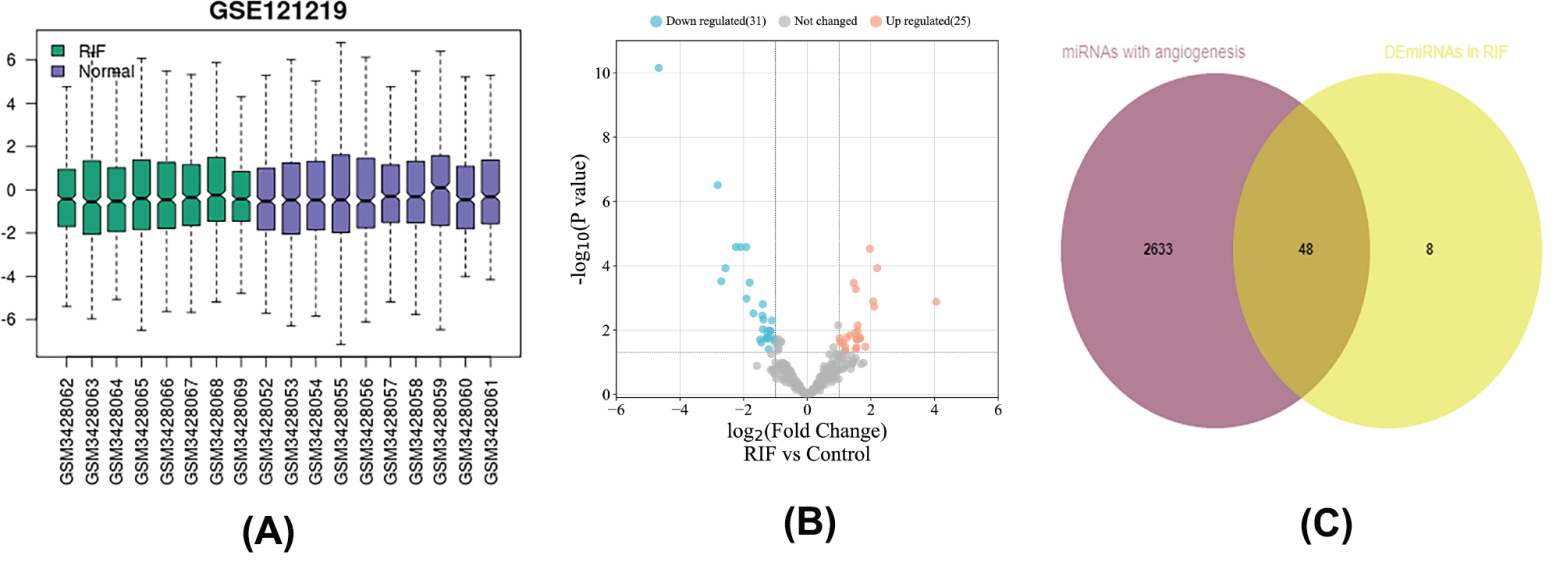
Boxplots, volcano plots and Venn diagram of miRNAs for GSE121219. (A) Boxplot of GSE121219. (B) Volcano plots of DEmiRNAs based on GSE121219. (C) Venn diagram of miRNAs for miRDIP and GSE121219, where the intersection section is predicted DEmiRNAs asscociated with angiogenesis in RIF. circRNA, circular RNA; miRNA, microRNA; RIF, recurrent implantation failure

**Fig. 4:**
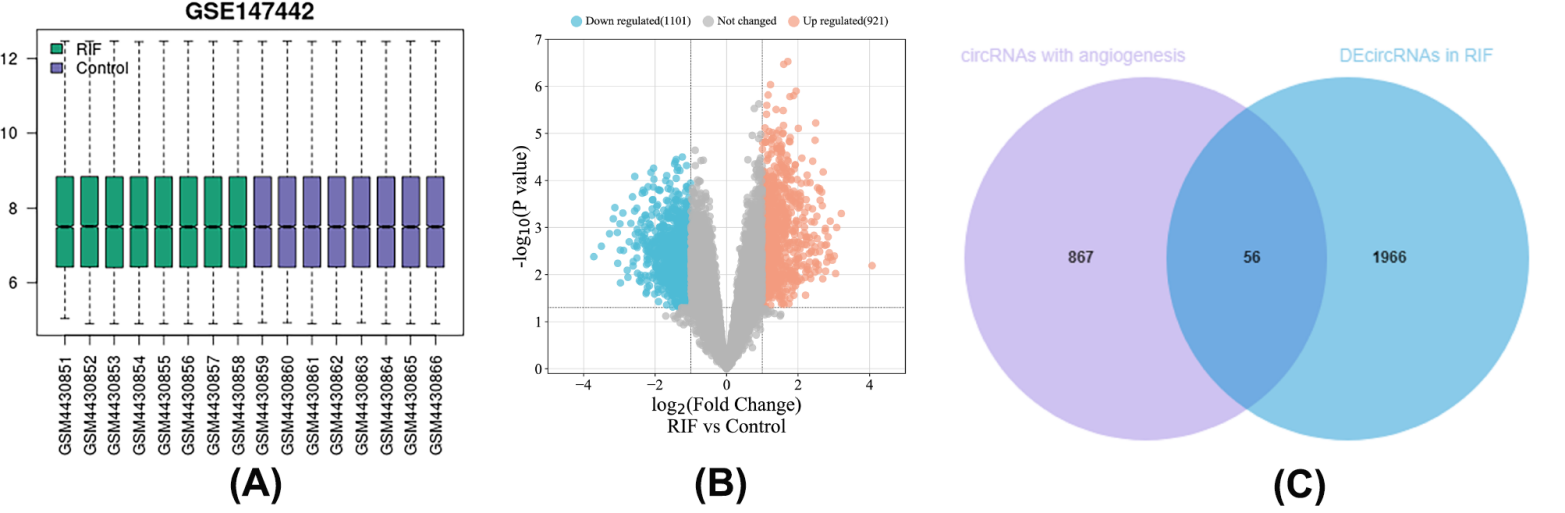
Boxplots, volcano plots and Venn diagram of circRNAs for GSE147442. (A) Boxplot of GSE147442 after standardization. (B) Volcano plots of DEcircRNAs based on GSE147442. (C) Venn diagram of miRNAs for starBase 2.0 and GSE147442, where the intersection section is predicted DEcircRNAs asscociated with angiogenesis in RIF. circRNA, circular RNA; miRNA, microRNA; RIF, recurrent implantation failure

A total of fifty-six DEmiRNAs (25 up-regulated and 31 down-regulated) were identified in the GSE121219 microarray (Figs. 3A and 4B, Supplementary Table 3). Based on the DEmRNAs associated with angiogenesis, two thousand, six hundred and eighty-one potential DEmiRNA targets were identified via miRDIP (Supplementary Table 4). Finally, forty-eight DEmiRNAs associated with angiogenesis were obtained after taking the intersection of the results from GSE121219 and the miRDIP (Fig. 3C).

### Prediction of angiogenesis-related circRNA-miRNA pairs

In total, two thousand and twenty-two DEcircRNAs (921 up-regulated and 1101 down-regulated) were obtained from the GSE147442 microarray (Fig. 4A and B, Supplementary Table 5). Based on the DEmiRNAs associated with angiogenesis, nine hundred and five potential DEcircRNA targets were identified from starBase 2.0 (Supplementary Table 6). Finally, after comparing the GSE147442 and starBase 2.0 datasets, 56 DEcircRNAs related to angiogenesis were obtained (Fig. 4C).

### Construction of the circRNA-miRNA‒mRNA network related to Angiogenesis

**Fig. 5:**
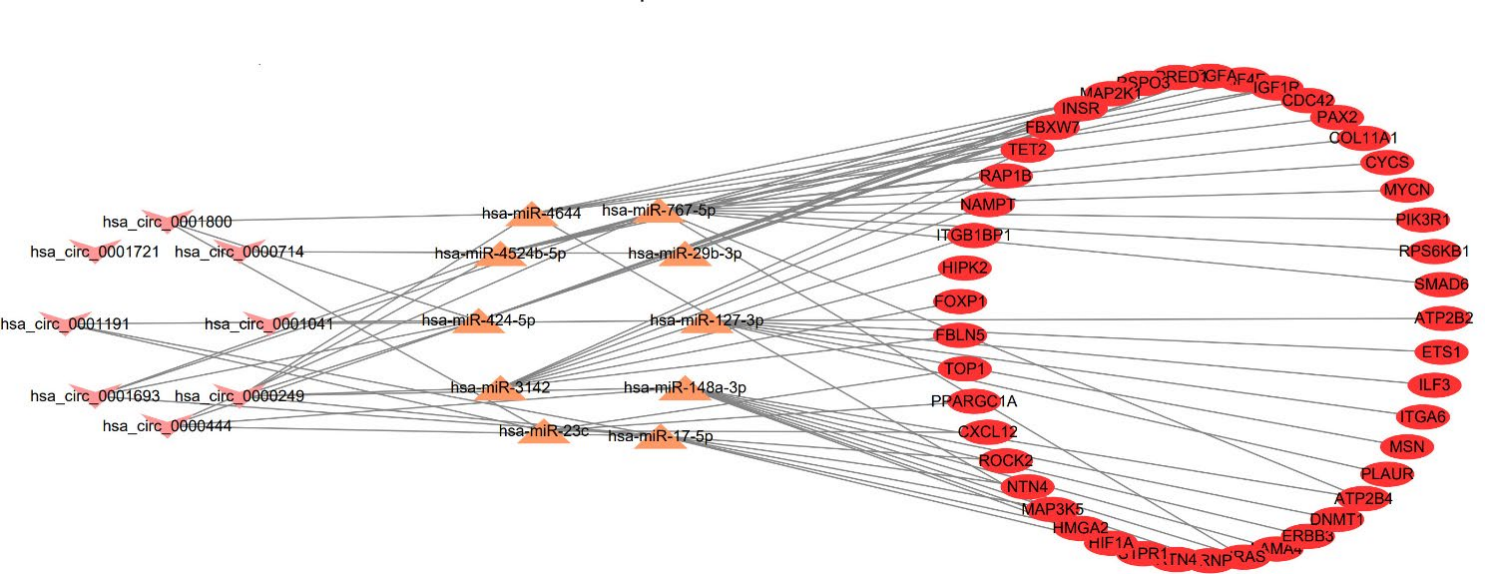
Figure 5 CircRNA-miRNA-mRNA regulatory network, which consists of 8 DEcircRNAs, 10 DEmiRNAs and 45 DERNAs. DEcircRNA, differentially expressed circular RNA; DEmiRNA, differentially expressed micro RNA; DERNA, differentially expressed RNA. Arrow-shape nodes: circRNAs, triangle nodes: miRNAs, ellipse-shaped nodes: mRNAs

After taking the intersection of DEmRNAs, DEmiRNAs and DEcircRNAs coexpressed with angiogenesis and removing nodes that had no connection to the central network, we obtained a circRNA-miRNA-mRNA regulatory network which is covering 45 DEmRNAs, 10 DEmiRNAs, and 8 DEcircRNAs. In this network, all DEcircRNAs, except hsa_circ_0001800, hsa_circ_0000714 and hsa_circ_0001721, were up-regulated in RIF. All DEmiRNAs, except hsa-miR-4644, hsa-miR-767-5p and hsa-miR-127-3p, were up-regulated(Fig. 5).

### Identification of hub genes in the CircRNA-miRNA‒mRNA regulatory network

**Fig. 6:**
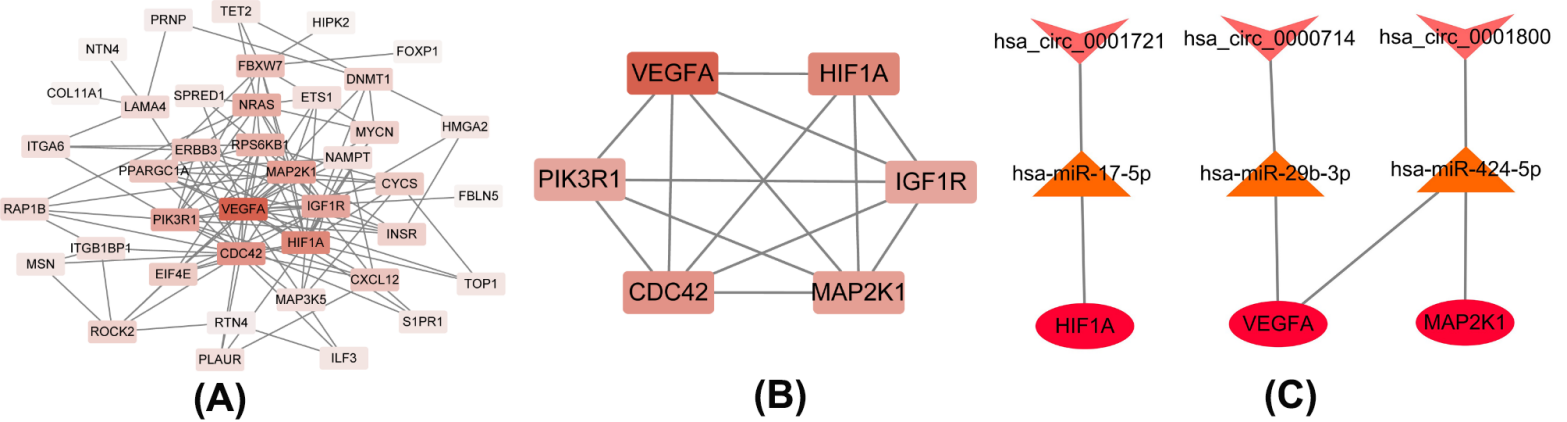
A PPI network and circRNA–miRNA–hub gene regulatory subnetwork. (A) A PPI network of the fifty-five target genes associated with angiogenesis in RIF. (B) Six hub genes extracted by cytoHubba plug-in. (C) CircRNA–miRNA–hub gene regulatory subnetwork, consisting of 3 circRNAs, 3 miRNAs, and 3 mRNAs. PPI, protein–protein interaction; circRNA, circular RNA; miRNA, microRNA; RIF, recurrent implantation failure

The potential interactions of the DEmRNAs were analyzed with the STRING database and visualized with Cytoscape 3.6.0 (Fig. 6A). The degree and closeness of the DEmRNAs were calculated by the cytoHubba plugin of Cytoscape (Supplementary Table 7). The top six hub DEmRNAs ranked by degree, betweenness centrality and closeness centrality, namely, vascular endothelial growth factor A (VEGFA), hypoxia inducible factor 1 subunit alpha (HIF1A), cell division cycle 42 (CDC42), mitogen-activated protein kinase kinase 1 (MAP2K1), insulin-like growth factor 1 receptor (IGF1R) and phosphoinositide-3-kinase regulatory subunit 1 (PIK3R1), were ultimately identified (Table 2; Fig. 6B).

However, based on the ceRNA hypothesis, the circRNA-miRNA-hub genes with a mode of down-up-down were chosen for further investigation. A circRNA-miRNA-hub genes regulatory network related to angiogenesis was constructed by Cytoscape 3.6.0 which included circRNAs, miRNAs, and mRNAs(Fig. 6C). In this hub network, all DEmRNAs were down-regulated. Hsa-miR-17-5p, hsa-miR-29b-3p and hsa-miR-424-5p were up-regulated. Hsa_circ_0001800, hsa_circ_0000714 and hsa_circ_0001721 were down-regulated in RIF.

**Table 2:** The top six genes obtained by the degree, betweenness, closeness

Name	Degree	Betweenness	Closeness
VEGFA	24	0.28	0.67
HIF1A	18	0.12	0.62
CDC42	16	0.22	0.60
MAP2K1	14	0.05	0.57
IGF1R	13	0.05	0.55
PIK3R1	13	0.07	0.57

### Gene ontology and Kyoto encyclopedia of hub DEmRNAs

**Fig. 7:**
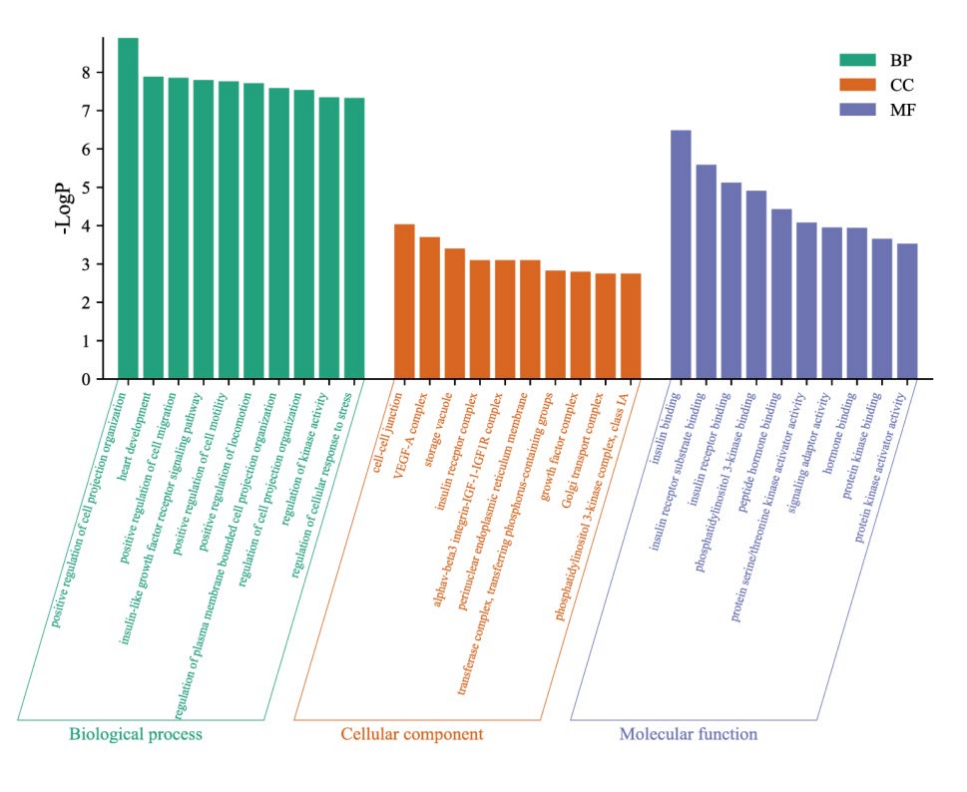
GO function analysis histogram. BP is marked by dark cyan, CC is marked by sienna and MF is marked by steel blue. The bar chart was constructed through the bioinformatics platform

GO enrichment analysis of six angiogenesis-related hub DEmRNAs, including biological process, cellular component, and molecular function, was performed (Fig. 7). KEGG pathway analysis was also performed to identify the signaling pathways associated with the six hub genes (Table 3), in which the “HIF-1 signaling pathway” was found to be the most relevant to angiogenesis in RIF.

**Table 3:** KEGG pathway analysis of six hub DEmRNAs

KEGG Pathway	*P*-value	Enrichment	HitGenes
HIF-1 signaling pathway	3.35E-12	230.75	HIF1A, IGF1R, PIK3R1,MAP2K1,VEGFA
Focal adhesion	7.44E-11	125.13	CDC42,IGF1R, PIK3R1,MAP2K1,VEGFA
Rap1 signaling pathway	9.28E-11	119.77	CDC42,IGF1R, PIK3R1,MAP2K1,VEGFA
Ras signaling pathway	1.67E-10	106.57	CDC42,IGF1R, PIK3R1,MAP2K1,VEGFA
VEGF signaling pathway	1.97E-10	341.04	CDC42,PIK3R1,MAP2K1,VEGFA
Autophagy - animal	6.8E-09	142.70	HIF1A, IGF1R, PIK3R1,MAP2K1
MAPK signaling pathway	1.3E-07	68.44	CDC42,IGF1R, MAP2K1,VEGFA
PI3K-Akt signaling pathway	2.74E-07	56.84	IGF1R, PIK3R1,MAP2K1,VEGFA
T cell receptor signaling pathway	7.89E-07	145.11	CDC42,PIK3R1,MAP2K1
FoxO signaling pathway	1.58E-06	115.20	IGF1R, PIK3R1,MAP2K1
mTOR signaling pathway	2.68E-06	96.74	IGF1R, PIK3R1,MAP2K1

### qRT‒PCR verification of the circRNA_0001721/miR-17-5p/HIF1A and circRNA_0000714/miR-29b-3p/VEGFA axes

According to the results of the GO and KEGG analyses, the HIF-1 signaling pathway was found to be the most relevant pathway to angiogenesis in RIF. We verified the relative gene expression levels of the key genes of the HIF-1 signaling pathway in the circRNA-miRNA‒mRNA regulatory network by qRT‒PCR. Through circRNA–miRNA–hub gene regulatory subnetwork (Fig. 6C), we have identified four potential regulatory axes: circRNA_0001721/miR-17-5p/HIF1A, circRNA_0000714/miR-29b-3p/VEGFA, circRNA_0001800/miR-424-5p/VEGFA and circRNA_0001800/miR-424-5p/MAP2K1. According to the ranking order of the six hub DEmRNAs, we choose the top two hub DEmRNAs, HIF1A and VEGFA for the further analysis(Table 2). Due to the significantly logFC value of circRNA 000714 compared to circRNA 0001800, in finally, we verified circRNA_0001721/miR-17-5p/HIF1A and circRNA_0000714/miR-29b-3p/VEGFA axes by qRT‒PCR.

**Fig. 8:**
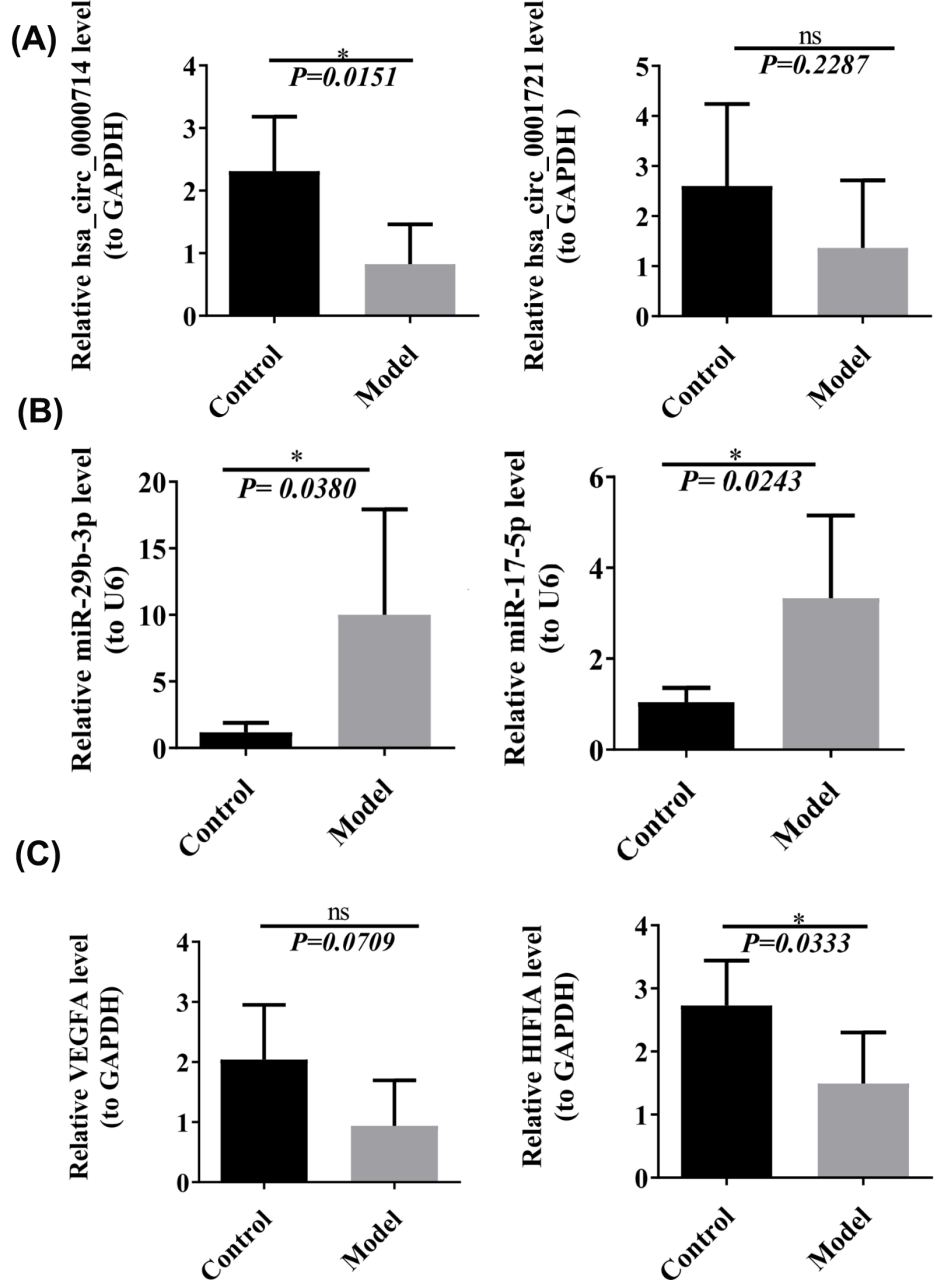
qRT-PCR validation. (A) The expression levels of CircRNA_0001721and circRNA_0000714. (B) The expression levels of miR-17-5p and miR-29b-3p. (C) The expression levels of HIF1A and VEGFA

Hsa_circ_0000714 and hsa_circ_0001721 were down-regulated, which was consistent with the microarray data (Fig. 8A). The qPCR results showed that the up-regulated expression levels of miR-29b-3p and miR-17-5p were generally consistent with the microarray results (Fig. 8B). The qRT‒PCR results also showed similar trends in the expression of VEGFA and HIF1A, which were also down-regulated, in the microarray analysis (Fig. 8C).

### Western blot verification of HIF1A and VEGFA

**Fig. 9:**
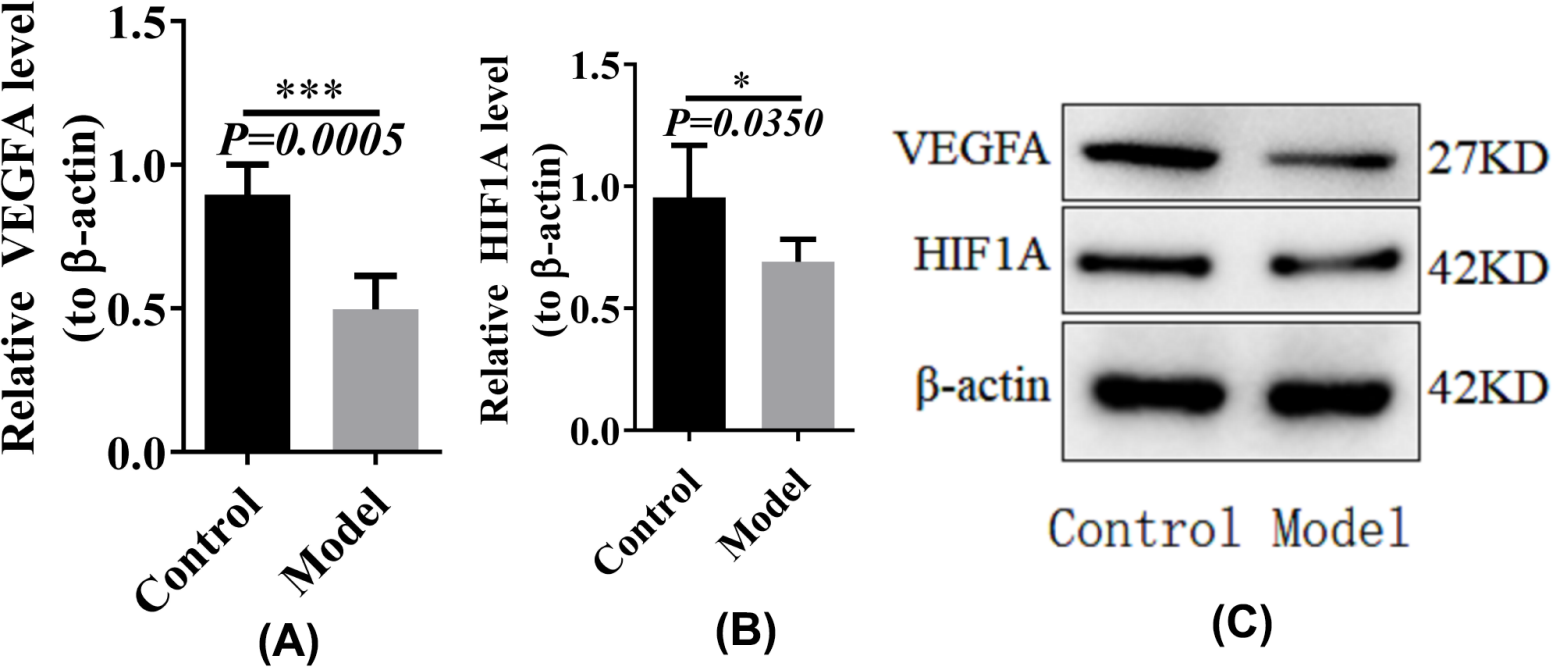
Western blot validation. (A) The relative levels of VEGFA. (B) The relative levels of HIF1A. (C) western blot image of HIF1A and VEGF. Full-length blots and gels are presented in Supplementary Figure and the grouping of gels/blots cropped from different gels

We used western blotting to verify the relative gene expression levels of genes involved in the HIF-1 signaling pathway. Both HIF1A and VEGFA were down-regulated in the model group, which was consistent with the microarray data Relative levels of VEGFA and HIF 1 A were down-regulated in the model group (Fig. 9A and B). The same results were also displayed the image of western blot (Fig. 9C).

## Discussion

Inadequate angiogenesis is known to be associated with RIF in both animal [25] and human studies [26]. The purpose of this study was to determine the ability of gene expression to improve endometrial angiogenesis during the diagnosis and treatment of RIF patients.

First, in this report, we identified one hundred and seventy-five DEmRNAs related to angiogenesis from the GSE11974 dataset. Among these DEmRNAs, forty-eight DEmRNAs and fifty-six DEcircRNAs related to angiogenesis were identified in the GSE103465 and GSE147442 datasets, respectively. We believe that changes in the expression of these genes may play an important role in the progression of endometrial angiogenesis in RIF. Soheila et al. provided evidence that ovarian stimulation and progesterone administration enhance endometrial angiogenesis through VEGF protein upregulation [27]. Furthermore, in addition to miR-16-5p, other miRNAs and molecules appear to be involved in angiogenic pathways, and further studies are needed [27]. Lin et al. demonstrated that the levels of miR-20a were up-regulated in endometriotic stromal cells and that the level of miR-20a was up-regulated by hypoxia inducible factor-1 (HIF-1). The upregulation of miR-20a caused the downregulation of dual specificity phosphatase-2 (DSP-2), which led to an increase in the expression of several angiogenic genes [28]. A number of reports revealed that noncoding RNAs and angiogenic genes were dysregulated in endometriosis and endometrial cancer and likewise played a key role in regulating embryo implantation [29–36]. However, the exact roles of circRNAs and their regulatory relationships with miRNAs and mRNAs associated with angiogenesis in RIF are still largely unknown.

Next, we sought to identify potential regulatory relationships between these DERNAs. We investigated the possible connections between the miRNAs and DEmRNAs and established two hundred and sixteen miRNA‒mRNA pairs in which forty-eight DEmiRNAs may modulate the expression of ninety-nine DEmRNAs. Then, we identified the upstream targeting circRNAs of forty-eight DEmRNAs in the miRNA‒mRNA network. Subsequently, we created a circRNA-miRNA‒mRNA regulatory network associated with angiogenesis in RIF by merging the circRNA-miRNA and miRNA‒mRNA pairs and removing the nodes without any connection to the central network.

The circRNA-miRNA‒mRNA regulatory network contains eight DEcircRNAs, namely, hsa_circ_0001800, hsa_circ_0000249, hsa_circ_0001693, hsa_circ_0001191, hsa_circ_0001721, hsa_circ_0000714, hsa_circ_0001041 and hsa_circ_0000444. However, none of the eight DEcircRNAs have been reported to regulate the angiogenic function associated with RIF. Additionally, we identified ten DEmiRNAs in the regulatory network. Dong et al. demonstrated that exosomal miR-424-5p inhibits primary granulosa cell proliferation and induces cellular senescence in polycystic ovary syndrome (PCOS) by blocking CDCA4-mediated Rb/E2F1 signaling [37]. Lin et al. reported that miR-17-5p and miR-424-5p were down-regulated and that VEGFA, IL-4, IL-6, and CA-125 were increased and inversely associated with miR-17-5p and miR-424-5p in endometriosis patients [38]. He et al. reported that estrogen induces epithelial–mesenchymal transition in endometriosis via circ0004712/miR-148a-3p sponge function [39]. There are no reports about these DEcircRNAs and DEmiRNAs involved in the regulation of endometrial angiogenesis related to the pathogenesis of RIF. Further studies should focus on the mechanism by which circRNAs serve as miRNA sponges to modulate endometrial angiogenesis to affect endometrial blood flow in RIF patients.

To further identify the key circRNAs involved in the regulatory network, we constructed a protein–protein interaction (PPI) network to screen the hub DEmRNAs. Six hub genes (VEGFA, HIF1A, CDC42, MAP2K1, IGF1R and PIK3R1) were identified for the construction of the hub circRNA regulatory network. Among the six hub DEmRNAs, serum VEGF was recognized as a marker of endometrial receptivity in infertile women [40]. However, Joachim et al. suggested that defects in the development of angiogenesis might provide an explanation for the endometrial factors that contribute to infertility, in which the lack of VEGF might lead to inadequate blood vessel growth [41]. Radia et al. revealed that VEGF expression was closely related to RIF [42]. Another report demonstrated that HIF-1α expression, microvessel density (MVD) and endometrial apoptosis were reduced in the peri-implantation endometrium of women with RIF, which suggested that altered endometrial HIF-1α expression and angiogenesis may contribute to implantation failure [43]. However, the roles of CDC42, MAP2K1, IGF1R and PIK3R1 in the regulation of angiogenesis-related RIF remain unknown, and findings on the role of these hub DEmRNAs in the regulation of vascular development during embryonic development may provide clues for the treatment of RIF. For instance, Christine et al. experimentally confirmed that CdGAP plays an important role in embryonic vascular development and VEGF-induced signaling by regulating CDC42 GTPase [44]. In addition, IGFBP-1 is secreted by predecidualized/decidualized endometrial stromal cells in the late secretory phase endometrium and pregnancy decidua. Thus, Rutanen suggested that IGFBP-1 has autocrine and paracrine functions in the regulation of endometrial proliferation and differentiation during embryo implantation [45]. Using MAP2K1, also termed a MEK2 inhibitor, Jing et al. suggested that decreasing the production of β-hCG in pregnant women could downregulate the expression of the VEGF-MEK/ERK signaling pathway, subsequently reducing angiogenesis and eventually leading to abnormal angiogenesis in villi [46]. PIK3R1 inhibits the PI3K signaling pathway, which controls important cellular activities, including protein synthesis, cell growth and proliferation, and angiogenesis, during embryonic development [47–49]. Based on the above findings, we hypothesize that these DEmRNAs may influence the occurrence of RIF by affecting endometrial vascularization during embryo implantation.

Finally, functional annotation and pathway analysis indicated that the six hub genes were involved in multiple cellular functions and signaling pathways. GO analysis revealed that these DEmRNAs were involved in biological activities such as positive regulation of cell projection organization, positive regulation of cell migration, insulin-like growth factor receptor signaling pathway and positive regulation of cell motility. In the cellular component category, the results revealed that these DEMs were mainly involved in the cell‒cell junction VEGF-A complex, storage vacuole, insulin receptor complex and α-β3 integrin-IGF-1-IGF1R complex. However, they were prominent in insulin binding, insulin receptor substrate binding, insulin receptor binding, phosphatidylinositol 3-kinase (PI3K) binding and peptide hormone binding at the cellular level. Pathway enrichment assessments revealed that the enriched pathways were the HIF-1 signaling pathway, focal adhesion pathway, Rap1 signaling pathway, Ras signaling pathway, VEGF signaling pathway, autophagy–animal pathway, MAPK signaling pathway, PI3K–Akt signaling pathway, T-cell receptor signaling pathway, FoxO signaling pathway and mTOR signaling pathway. Among them, the ERK/MAPK signaling pathway can induce epithelial cell proliferation and stromal cell decidualization by regulating various growth factors, immune factors, and hormones, playing a crucial role in embryo implantation [50]. PI3K/Akt signaling pathway and FoxO signaling pathway are involved in regulating the growth of oocytes, the development of primordial follicles, and the proliferation and differentiation of granulosa cells [51].

Based on the above results, we speculated that the HIF-1 signaling pathway might play a vital role in endometrial angiogenesis in RIF and be closely associated with angiogenesis [52, 53]. Research has confirmed that under hypoxic conditions, HIF-1α is activated and regulates transcription factors such as VEGF to participate in angiogenesis [54–56]. The mechanism may involve hypoxia activating the PI3K/AKT pathway and preventing posttranslational hydroxylation and subsequent degradation of HIF-1α. Upon HIF-1α accumulation, HIF-1α is transferred to the nucleus and forms a transcription initiation complex, which initiates the transcription of target genes, resulting in an increase in the levels of corresponding protein products, including VEGF, thus promoting angiogenesis [57]. Yokoe [58] and others also showed that HIF-1α could stably exist in a hypoxic environment and regulate the synthesis of VEGF protein through the PI3K pathway and hypoxia-activated PI3K/Akt/mTOR pathway [59]. Recent studies have also shown that under hypoxic conditions, HIF-1α could directly or indirectly promote the expression of VEGF, activate its receptor, and participate in the regulation of angiogenesis through VEGF [60, 61], while the endometrium was in a physiologically hypoxic environment during embryo implantation [62].

Based on the established circRNA-miRNA‒mRNA regulatory axis of RIF associated with angiogenesis and the effect of the HIF-1 signaling pathway on angiogenesis in the RIF endometrium, we assume that the hsa_circ_0001721/hsa-miR-17-5p/HIF1A axis is at the core of the pathogenesis of RIF and that hsa_circ_0001721 acts as a sponge for hsa-miR-17-5p due to a decrease in hsa_circ_0001721 expression in the RIF endometrium, which may weaken the adsorption of hsa-miR-17-5p and cause a decrease in HIF1A expression, resulting in a decrease in endometrial angiogenesis, thus promoting the progression of RIF. In both cases, we hypothesized that the hsa_circ_0000714/hsa-miR-29b-3p/VEGFA axis has a similar mechanism. Through the use of the RIF mouse model, we validated by qRT‒PCR that hsa_circ_0000714 and hsa_circ_0001721 were down-regulated, miR-29b-3p and miR-17-5p were up-regulated, and VEGFA and HIF1A were down-regulated. Additionally, the protein levels of VEGFA and HIF1A were down-regulated in the RIF model group, which was consistent with the microarray data.

To the best of our knowledge, the present study is the first attempt to construct a circRNA-miRNA‒mRNA network and determine the pathways with the greatest potential for regulating the pathogenesis of endometrial angiogenesis in RIF. However, our research has several limitations that we should consider, including the limitations of the mouse model that we should use clinical tissue samples for further validation.

## Conclusions

We identified DEmRNAs associated with endometrial angiogenesis and constructed a circRNA-miRNA‒mRNA regulatory network based on these DEmRNAs. In addition, six hub DEmRNAs were identified through protein–protein interaction (PPI) analysis to establish the central circRNA regulatory network. The HIF-1 signaling pathway, which is related to RIF endometrial angiogenesis, was the most influential pathway in the network. In conclusion, our findings indicated that the CircRNA_0001721/miR-17-5p/HIF1A and circRNA_0000714/miR-29b-3p/VEGFA axes might play a role in the pathogenesis of endometrial angiogenesis in RIF.

### Electronic supplementary material

Below is the link to the electronic supplementary material.

Supplementary Material 1

Supplementary Material 2

Supplementary Material 3

Supplementary Material 4

Supplementary Material 5

Supplementary Material 6

Supplementary Material 7

Supplementary Material 8

## Data Availability

Data is provided within the manuscript or supplementary information files.

## Abbreviations

RIF: Recurrent implantation failure
FET: Freeze-thaw embryo transfer
circRNA: Circular RNA
miRNA: microRNA
DEcircRNAs: Differential expression of circRNAs
DEmiRNAs: Differential expression of miRNAs
DEmRNAs: Differential expression of mRNAs
GEO: The Gene Expression Omnibus
PPI: Protein-protein interaction
GO: Gene Ontology
KEGG: Kyoto Encyclopedia of Genes and Genomes
qRT-PCR: Quantitative real time polymerase chain reaction
VEGFA: Vascular endothelial growth factor A
HIF1A: Hypoxia inducible factor 1 subunit alpha
CDC42: Cell division cycle 42
MAP2K1: mitogen-activated protein kinase kinase 1
IGF1R: Insulin like growth factor 1 receptor
PIK3R1: Phosphoinositide-3-kinase regulatory subunit 1
